# Cryo-electron microscopy and image classification reveal the existence and structure of the coxsackievirus A6 virion

**DOI:** 10.1038/s42003-022-03863-2

**Published:** 2022-09-02

**Authors:** Carina R. Büttner, Radovan Spurný, Tibor Füzik, Pavel Plevka

**Affiliations:** Central European Institute of Technology - Masaryk University, Structural Biology, Structural Virology, Kamenice 5, 62500 Brno, Czech Republic

**Keywords:** Virus structures, Cryoelectron microscopy

## Abstract

Coxsackievirus A6 (CV-A6) has recently overtaken enterovirus A71 and CV-A16 as the primary causative agent of hand, foot, and mouth disease worldwide. Virions of CV-A6 were not identified in previous structural studies, and it was speculated that the virus is unique among enteroviruses in using altered particles with expanded capsids to infect cells. In contrast, the virions of other enteroviruses are required for infection. Here we used cryo-electron microscopy (cryo-EM) to determine the structures of the CV-A6 virion, altered particle, and empty capsid. We show that the CV-A6 virion has features characteristic of virions of other enteroviruses, including a compact capsid, VP4 attached to the inner capsid surface, and fatty acid-like molecules occupying the hydrophobic pockets in VP1 subunits. Furthermore, we found that in a purified sample of CV-A6, the ratio of infectious units to virions is 1 to 500. Therefore, it is likely that virions of CV-A6 initiate infection, like those of other enteroviruses. Our results provide evidence that future vaccines against CV-A6 should target its virions instead of the antigenically distinct altered particles. Furthermore, the structure of the virion provides the basis for the rational development of capsid-binding inhibitors that block the genome release of CV-A6.

## Abstract (web summary)

A cryo-EM structure for the three conformers of coxsackievirus A6 provides insight into the infection process of this enterovirus, which is responsible for numerous cases of hand, foot, and mouth disease.

## Introduction

Enteroviruses are a globally distributed and ubiquitous threat to humans and livestock, causing infections of the respiratory and gastrointestinal tracts, skin, and central nervous system^1–3^. In recent years, Coxsackievirus A6 (CV-A6) has overtaken enterovirus A71 (EV-A71) and Coxsackievirus A16 (CV-A16) as the major causative agent of hand, foot and mouth disease (HFMD) in young children^4^. The virus is also emerging as a frequent causative agent of HFMD in adolescents^5^. Unlike the classical HFMD viruses EV-A71 and CV-A16, CV-A6 is associated with atypical HFMD, which manifests as systemic large vesicular eruptions and onychomadesis or orchiepididimitis, but can also cause aseptic meningitis or encephalomeningitis^4,6–9^. Common in Southeast Asia, where it has been the principal cause of HFMD since the early 2010s^10–14^, CV-A6 has since spread globally^15–21^. Strains of CV-A6 that cause severe clinical symptoms in adults have been identified^19,22^. Available HFMD treatments and vaccines are predominantly directed against EV-A71 and CV-A16, whereas no vaccine exists to counter CV-A6.

Picornaviruses, including viruses from the genus *Enterovirus* such as CV-A6, CV-A16 and EV-A71, are small, non-enveloped icosahedral viruses with single-stranded positive sense RNA genomes (reviewed in ref. ^23^). To initiate infection, enterovirus particles progress through a set of distinct functional intermediates: the infectious native virion expands into a destabilised altered or A-particle, in which the internal capsid protein VP4 has been displaced^24^. The genome is released through a capsid opening, leaving the empty capsid as the non-infectious end product^25–28^. Although high-resolution structures of the EV-A71 and CV-A16 virions are available^29–31^, the structural characterisation of CV-A6 is limited to altered and empty particles and virus-like particles^32,33^. The absence of virions in CV-A6 preparations led Xu et al. (2017) to speculate that the altered particle is the sole infectious species of CV-A6 and an exception to the canonical enterovirus infection pathway^32^.

Here we report the structure of the previously unrecognised CV-A6 virion. We determined cryo-EM structures of the compact CV-A6 virion, the expanded altered particle, and the empty particle to a better than 3-Å resolution. We used a fluorescence-based infection assay to show that CV-A6 has an infectious unit-to-particle ratio of 1: 500. Our findings reestablish the canonical steps of the enterovirus infection process for CV-A6 and enable the rational design of capsid-binding therapeutics against CV-A6.

## Results and discussion

### CV-A6 virion, altered, and empty particles

**Fig. 1:**
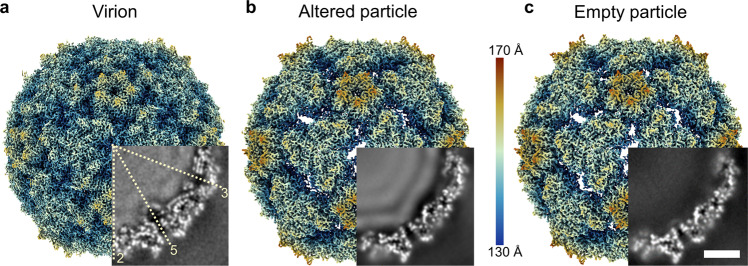
Structure of CV-A6 virion, altered, and empty particle. Cryo-electron density map of CV-A6 virion (**a**), altered particle (**b**), and empty particle (**c**), viewed along the twofold icosahedral symmetry axis, coloured according to distance from centre of particle. The insets show central slices through the particle interior (central slice, thickness 5 Å). Virion and altered particle contain genomic RNA (**a**, **b**). Positions of icosahedral symmetry axes are indicated by dashed lines in **a**. Scale bar 5 nm.

The sample of purified CV-A6 contained a mixture of genome-containing and empty particles (Fig. 1 and Supplementary Fig. 1a). The combination of two-dimensional (2D) and 3D classification identified 1769 virions (1.2%) in 131,286 images of altered particles with expanded capsids (91.4%) and 10,613 empty particles (7.4%) (Supplementary Fig. 2). The structure of the CV-A6 virion was determined to a resolution of 2.68 Å, whereas those of the altered and empty particles reached resolutions of 2.50 and 2.82 Å, respectively (Fig. 1, Table 1 and Supplementary Fig. 2d–f). Overall, the CV-A6 virion exhibits surface features typical for the virions of enteroviruses, including the prominent star-shaped plateau (mesa) around each fivefold symmetry axis, a pronounced depression (canyon) encircling the mesa, and three-bladed propeller shapes around the threefold symmetry axes (Fig. 1). The virion is 3.2% smaller than the altered and empty particles, a similar change in size to that observed for other enteroviruses such as EV-A71, CV-A16, CV-B3, and poliovirus 1^30,31,34,35^. The virion has less pronounced surface features (Fig. 1a–c). Unlike the altered and empty particles, the CV-A6 virion does not contain openings located on the twofold symmetry axes of its capsid (Fig. 1a). The smaller radius and the absence of open channels in the compact structure of the CV-A6 virion provide evidence that it represents the genuine native conformation of a mature enterovirus.

**Table 1:** Cryo-EM data collection, refinement and validation statistics.

	CV-A6 virion	CV-A6 altered particle	CV-A6 empty particle
*EMDB ID*	EMD-14186	EMD-14183	EMD-14184
*PDB ID*	7QW9	7QVX	7QVY
**Data collection and processing**			
Microscope	Titan Krios	Titan Krios	Titan Krios
Detector	Falcon II	Falcon II	Falcon II
Magnification	75,000	75,000	75,000
Voltage (kV)	300	300	300
Electron dose (e^—^/ Å)	45	45	45
Defocus range (µm)	−3.0 to −1.0	−3.0 to −1.0	−3.0 to −1.0
Pixel size (Å)	1.063	1.063	1.063
Symmetry imposed	I4	I4	I4
Initial particle images (no.)	211,991	211,991	31,064
Final particle images (no.)	1769	131,286	10,613
Map resolution (Å)	2.68	2.50	2.82
FSC threshold	0.143	0.143	0.143
Map resolution range (Å)	2.3 - ∞	2.3 - ∞	2.3 - ∞
**Refinement**			
Initial model used (PDB)	5XS4	5XS4	5XS4
Model resolution	2.84	2.52	2.82
FSC threshold	0.5	0.5	0.5
Map-to-model correlation coefficient	0.90	0.87	0.87
Map-sharpening B-factors (Å^2^)	locally sharpened	−75	−70
***Model composition***			
Non-hydrogen atoms ^a^	6530	5159	5028
Ligand atoms	20 (STE); 16 (MYR)	-	-
***B factors*** **(Å**^**2**^**)**			
Protein	66.0	29.0	37.0
Ligand	104 (STE); 138 (MYR)	-	-
***R.m.s.deviations***			
Bond lengths (Å)	0.0121	0.0091	0.0099
Bond angles (°)	1.5715	1.2290	1.3057
***Validation***			
Clash score	5.28	3.93	4.04
Molprobity score	1.53	1.18	1.30
Poor rotamers (%)	0	0	0
***Ramachandran plot***			
Favoured (%)	97.89	98.15	97.45
Allowed (%)	2.11	1.85	2.55
Disallowed (%)	0	0	0

^a^ Icosahedral asymmetric unit.

The structures of the altered and empty CV-A6 particles reported here are similar to those of the altered particle (Protein Data Bank (PDB) entry 5XS4) and procapsid (5XS5) reported previously^32^. The corresponding maps can be superimposed with correlation coefficients of 0.94 and 0.95, and the protomer structures with an overall root mean square deviation (r.m.s.d.) of 0.73 and 0.75 Å, respectively, matching 612 of 623 and 594 of 610 C^α^ atoms available for the comparisons (Supplementary Fig. 3 and Supplementary Table 1). The similarity is expected for closely related virus strains Gdula, used in this study, and TW-2007-00141 which share 94, 96, and 96% sequence identity for VP1, VP2, and VP3, respectively (Supplementary Fig. 4).

### Structure of the CV-A6 virion

**Fig. 2:**
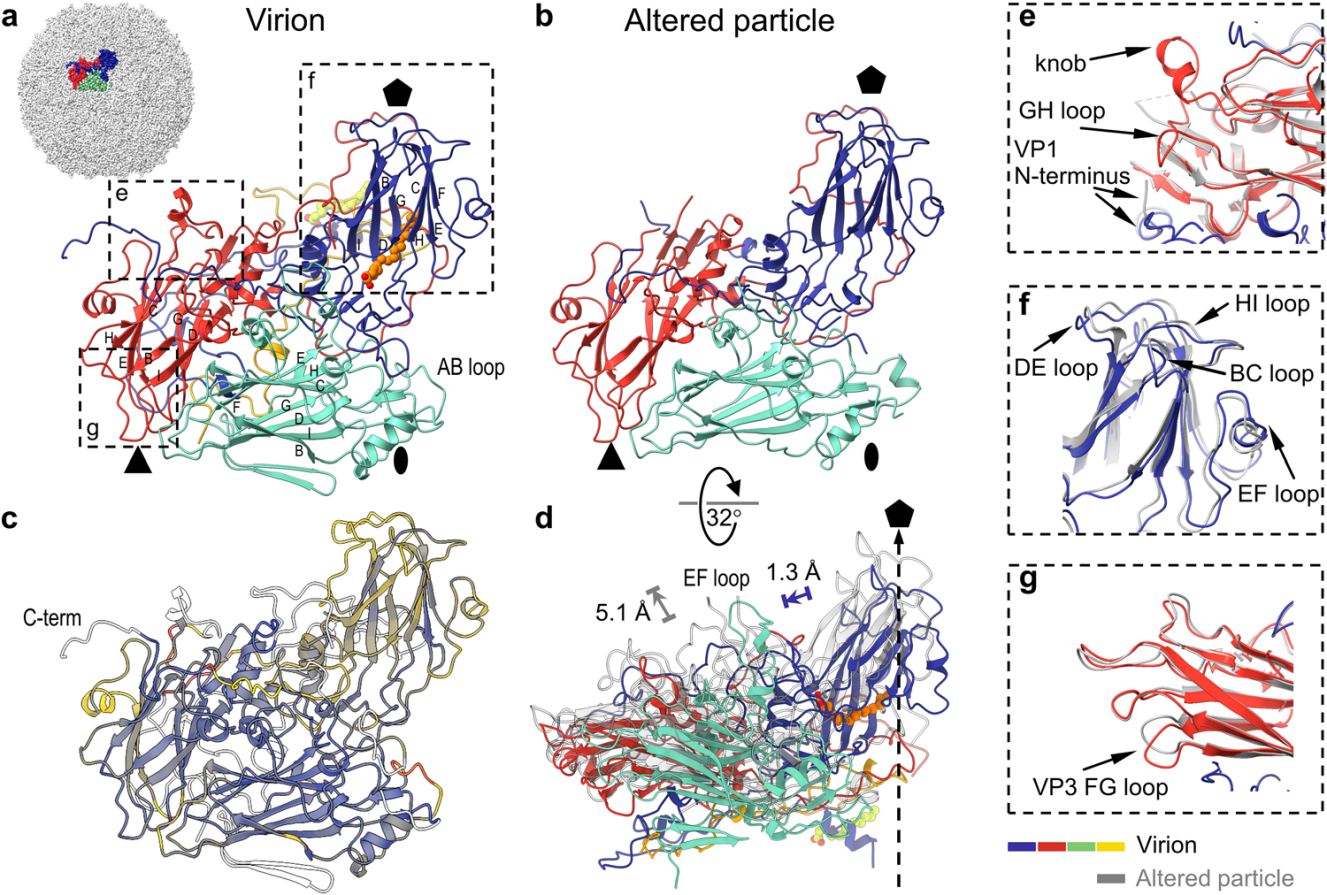
Structures of icosahedral asymmetric units of CV-A6 virion and altered particle. **a** Ribbon representation of icosahedral asymmetric unit of CV-A6 virion with VP1 coloured in blue, VP2 in green, VP3 in red, and VP4 in yellow. The relative position of the depicted protomer in the virion is indicated in the inset. Positions of the twofold, threefold, and fivefold icosahedral symmetry axes are indicated with an oval, triangle, and pentagon, respectively. The pocket factor (stearic acid) and the putative myristate attached to the N-terminus of VP4 are shown as ball-and-stick models in orange and yellow-green, respectively. Beta-strand identifiers of the jelly roll CHEF and BIDG sheets are indicated. The dashed rectangles highlight the structural details shown in **e**–**g**. **b** Icosahedral asymmetric unit of altered particle, shown in same orientation as in **a**. **c** Structural deviations between virion and altered particle. R.m.s. deviations are mapped onto the protomer of the virion. Regions of low deviation (r.m.s.d. ≤3 Å) are coloured in blue, through yellow (≤4 Å), to orange (≤6 Å) and dark red (>6 Å). Chain segments missing in the altered particle are coloured white. **d** Side view of the protomers from virion and altered particle. In the virion, the centre of mass of the jelly roll core of VP1 is shifted 1.3 Å closer to the centres of mass of VP2 and VP3 than in the altered particle. Upon expansion, the centre of mass of the altered particle protomer shifts by 5.1 Å. The centre of mass of the virion was calculated using an equivalent subunit and amino acid selection that matches the protomer of the altered particle. **e**–**g** Details of major structural differences between virion and altered particle. The virion protein chains are shown in colours, those of the altered particle in grey.

The capsid of the CV-A6 virion is built from 60 protomers composed of subunits VP1, VP2, VP3, and VP4 (Fig. 2a, b). The major capsid proteins VP1, VP2, and VP3 are arranged with pseudo-*T* = 3 icosahedral symmetry with VP1 subunits forming pentamers around the fivefold axes and VP2 and VP3 forming heterohexamers around the threefold symmetry axes of the capsid. VP4 are 69-residue long peptides attached to the inner face of the capsid. The cryo-EM density map of the CV-A6 virion provides evidence that VP3 contains one additional C-terminal residue (Asn241) (Supplementary Fig. 5) than originally annotated^36^, implying that the cleavage between VP3 and VP1 occurs at a downstream cleavage site. We have accordingly added Asn241 to VP3 and renumbered the VP1 amino acid sequence from Asp1 to Phe304 (Supplementary Fig. 4). The cryo-EM reconstruction enabled the building of residues 9 to 300 of 304 of VP1, 7 to 256 of 256 of VP2, the complete structure of 241 residues of VP3, and residues 15 to 69 of 69 of VP4 (Fig. 2 and Supplementary Fig. 6). VP1, VP2 and VP3 have the single jelly roll fold that is characteristic of picornaviruses and many other viruses with icosahedral capsids (Fig. 2a, b and Supplementary Fig. 6). The β-sandwich of the jelly roll fold is composed of two antiparallel four-stranded β-sheets named according to convention BIDG and CHEF^37,38^. The β-strands are connected by loops with variable lengths, which are named after their flanking strands (Fig. 2a, d–g and Supplementary Figs. 4,  6). The structured part of VP4 does not contain any secondary structure elements except for a short α-helix formed by residues 51-54. VP4 interacts with all three major capsid proteins. Almost all side chains were clearly resolved in the cryo-EM map of the CV-A6 virion (Supplementary Fig. 1g). The resolution of the cryo-EM map of the virion varied from 2.4 to 11 Å (Supplementary Fig. 1b, c). The surface-exposed C-terminal four residues of VP1, the EF loop (residues 138–145) of VP2, and residues 15 to 23 of VP4 are less well resolved, suggesting they are flexible (Fig. 2a, c, d, Supplementary Fig. 6 and Supplementary Table 2).

The protomer of capsid proteins forming the CV-A6 virion includes the interfaces VP1:VP3 with a buried surface area of 4050 Å^2^, VP1:VP2 with a buried surface area of 2100 Å^2^, VP2:VP3 with a buried surface area of 1600 Å^2^, and VP1:VP4 with a buried surface area of 1350 Å^2^. The protomer is stabilised by 237 hydrogen bonds and 15 salt brides, of which two are also conserved in CV-A16 and EV-A71 (VP3 H35:VP2 E37 and VP3 D18:VP1 R248)^33^. The genesis of a pentamer, a speculated assembly intermediate of enterovirus capsids, from protomers involves the formation of interfaces with a buried surface area of 10,100 Å^2^ per protomer.

**Fig. 3:**
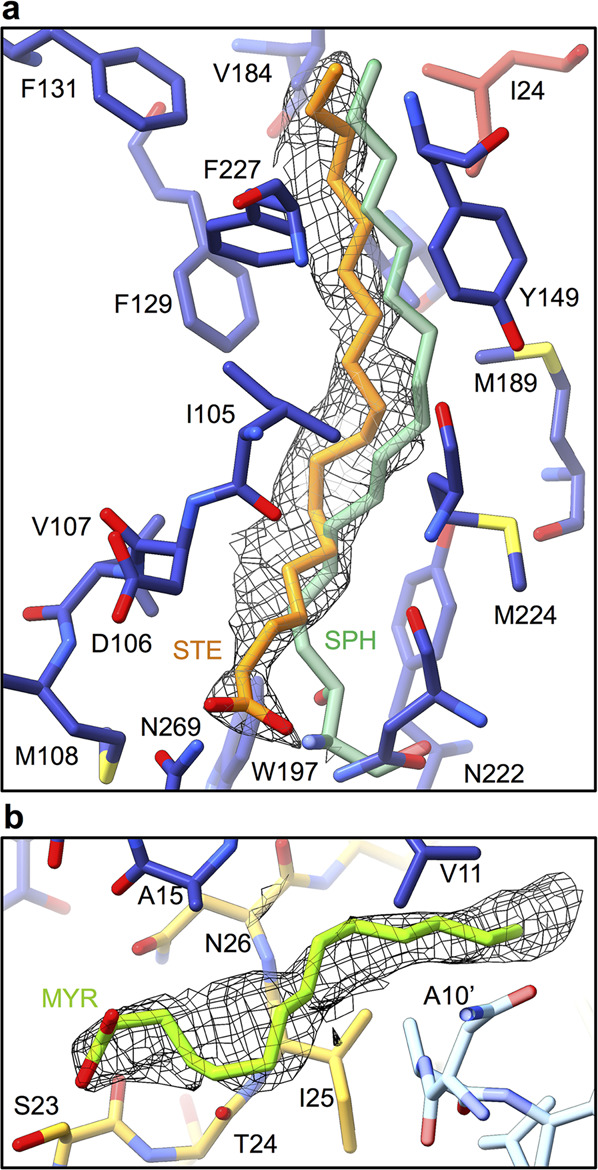
Pocket factor and myristate in CV-A6 virion. **a** Pocket factor binding site in VP1 pocket of CV-A6. The proteins and lipid are shown in stick representation. Residues of VP1 are shown with carbon atoms coloured in blue, VP3 in red, and the CV-A6 pocket factor stearic acid in orange. For comparison, the pocket factor of poliovirus 1, sphingosine (PDB 1ASJ)^39^, is shown in light green. The density of the CV-A6 pocket factor is shown as a grey mesh. **b** Putative myristate attached to N-terminus of VP4 is shown in green. The density of the myristate is depicted as a grey mesh. The myristate interacts with residues of two VP1 subunits (blue, light blue) and VP4 (yellow).

The β-sandwich of VP1 contains a central hydrophobic cavity, called a pocket, located beneath the floor of the canyon (Fig. 2a). The pocket is a feature shared by virions of most enteroviruses (reviewed in ref. ^23^). The pocket of CV-A6 contains an elongated density (pocket factor), which we modelled as C-18 stearic acid in accordance with the length of the electron density in the locally sharpened and masked map (Fig. 3a). The density distribution calculated from the stearic acid structure has a correlation coefficient with the corresponding part of the cryo-EM map of 0.82. The pocket factor of CV-A6 interacts with 19 amino acids (Supplementary Table 3 and Fig. 3a). The position of the stearic acid in the VP1 pocket of CV-A6 is similar to that of the sphingolipid in poliovirus (Fig. 3a) (PDB 1ASJ^39^). The cryo-EM density of the stearic acid in CV-A6 is discontinuous in the sharpened map, whereas it is well-defined and continuous in the locally sharpened map but has lower density values than the surrounding protein structure, indicating possible flexibility of the pocket factor, variability in the type of the molecule, or lower than 100% occupancy. The majority of virions of enteroviruses structurally characterised to date possess a hydrophobic pocket in the VP1 subunit, containing a pocket factor. In contrast, no pocket factors have been found in altered and empty particles. Pocket factor expulsion seems to be a prerequisite of enterovirus genome release^30,40–42^. The pocket factor has been suggested to stabilise the mature virion during transport to a new host or cell^42^. Therefore, the presence of the pocket factor provides further evidence that the structure represents the virion of CV-A6.

The N-terminal arm of VP1 in the CV-A6 virion (residues 9 to 43) extends towards the fivefold symmetry axis on the inside of the capsid and interacts with residues 25–30 of VP4 from the same protomer (Fig. 2a, d). The cryo-EM map of the CV-A6 virion contains an elongated continuous density positioned parallel to the N-terminal segment of VP4 (Fig. 2a, d). The elongated density interacts mostly with hydrophobic side chains of VP1 and VP4 (Val11, Leu14, Ala15 of VP1; Ser23, Thr24, Ile25, Asn26 of VP4; Ala10, Thr13 of neighbouring VP1 subunit; Phe27 of neighbouring VP4 subunit; cut-off range 4.7 Å). We speculate that this density corresponds to myristate attached to an N-terminal glycine of VP4 (Fig. 3b)^43,44^.

### Structural differences between the CV-A6 virion and altered and empty particles

The structures of the altered and empty particles of CV-A6 are similar, and the capsid proteins forming their icosahedral asymmetric units can be superimposed with an r.m.s.d. of 0.31 Å for 633 of 638 C^α^ atoms available for the comparison (Supplementary Table 1 and Supplementary Table 2). The buried surface area of 4400 Å^2^ per protomer within the altered capsid is less than half than that of the virion. Unstructured surface loops in the altered and empty particles yield less reliable estimates of maximal particle radii of 167 and 168 Å, respectively. The maximum radius of the CV-A6 virion is 161 Å. Like the previously studied enteroviruses, the conversion of CV-A6 virions to the altered particles is connected to the loss of ordered structures of VP4 subunits and the N- and C-termini of the major capsid proteins, but also of surface loops of VP1, VP2, and VP3. The structures of the altered particle and the virion of CV-A6 can be superimposed with an r.m.s.d. of 0.82 Å for 548 C^α^ atoms of 659 available for the comparison (Fig. 2c, e–g, Supplementary Fig. 3 and Supplementary Table 1). The centre of mass of the protomer in the expanded altered particle is 135 Å from the particle centre, whereas it is 130 Å in the virion. The conformational transition from virion to altered particle results in an absolute shift of 5.1 Å in the protomer centre of mass (Figs. 1a, b,  2d). Local differences between the altered particle and the CV-A6 virion are found in the surface-exposed loops of VP1 located close to the fivefold symmetry axes. Moreover, the core of VP1 in the altered particle is positioned closer to VP2 and VP3 than in the virion (Fig. 2d). The GH loop of VP1 (residues 205–214), the EF loop of VP2 (139–143), and the GH loop of VP3 (175–186) are structured in the virion but not in the altered particle (Fig. 2c, e and Supplementary Fig. 3). In the altered particle, the EF loop of VP1 is bent 0.47 Å towards the particle centre relative to its position in the virion (Fig. 2f and Supplementary Fig. 3). The externalisation of the N-terminus of VP1 in the altered particle induces displacement of residues 169–175 from the GH loop of VP3, which change their conformation from an α-helix to a β-strand (res. 170–172) (Fig. 2e).

In the empty particle, the side chain of Trp109, located at the interprotomer contact near the twofold symmetry axis, adopts two alternative side-chain conformations related by a 180˚ flip (Supplementary Fig. 7a). The rotamers (p90 and p-90), which occur in the protein databank with 6 and 11% frequency, respectively, have temperature factors of 17 and 19 Å^2^ in the structure of the CV-A6 empty particle. The bulky indole ring of the tryptophan side chain populates a primary rotamer in >85% of cases, and its rotamer switching is strongly controlled by solvent exposure^45^. Trp109 is buried in the altered particle, where it adopts only one rotamer (Supplementary Fig. 7c), yet becomes partially solvent-exposed in the empty particles which allows it to exist in an approximately 50:50 occupancy ratio of the two indole ring-flipped conformations. The occurrence of the alternative side-chain conformations of Trp109 is further enabled by the flexibility of the interprotomer contacts in the empty CV-A6 particle (Supplementary Fig. 7b). Trp109, which is strictly conserved among enteroviruses, has not been previously modelled with alternative conformations, but both rotamers were observed in different structures, e.g., p-90 in the empty particles of CV-A16 (PDB 5C9A), D68 (6AJ3), poliovirus 1 (1POV), and previously reported CV-A6 (5XS5), whereas the p90 conformer is found in the empty particles of CV-10 (6ACW) and EV-A71 (4GMP). Occasionally, the side chain of another amino acid of VP2 or VP3 reaches into the space taken by the p90 conformer, as occurs in CV-A16 (5C9A) or enterovirus D68 (6AJ3). This is also the case in the altered particle of CV-A6, where the side chain (atom CD1) of Ile170 from VP3 reaches into the volume of the p-90 conformer (Supplementary Fig. 7a, c). In contrast, in the empty particle Ile170 has moved away as a result of the disordered VP3 GH loop (Supplementary Fig. 7b).

Residues 45–47 from the N-terminal AB loop of VP2 form a short α-helix located near the twofold symmetry axis of the altered particle, whereas in the empty particle these residues are disordered (Fig. 2a, b and Supplementary Fig. 7b, d). The α-helix is not resolved in the previously published structure of the altered particle of CV-A6 (5XS4^32^), however, similar α-helical elements are present in altered particles of CV-A10 (6AKT) and CV-A16 (4JGY). Because of its position, the α-helix may stabilise the altered particle prior to genome release. The observed structural differences between the altered and empty particles indicate that in the absence of the genome the interprotomer contacts are weaker, thereby allowing greater conformational transitions of surface-exposed loops, secondary structure elements, and side chains.

### Antigenic properties of virions of CV-A6 and other enteroviruses causing hand, foot, and mouth disease

**Fig. 4:**
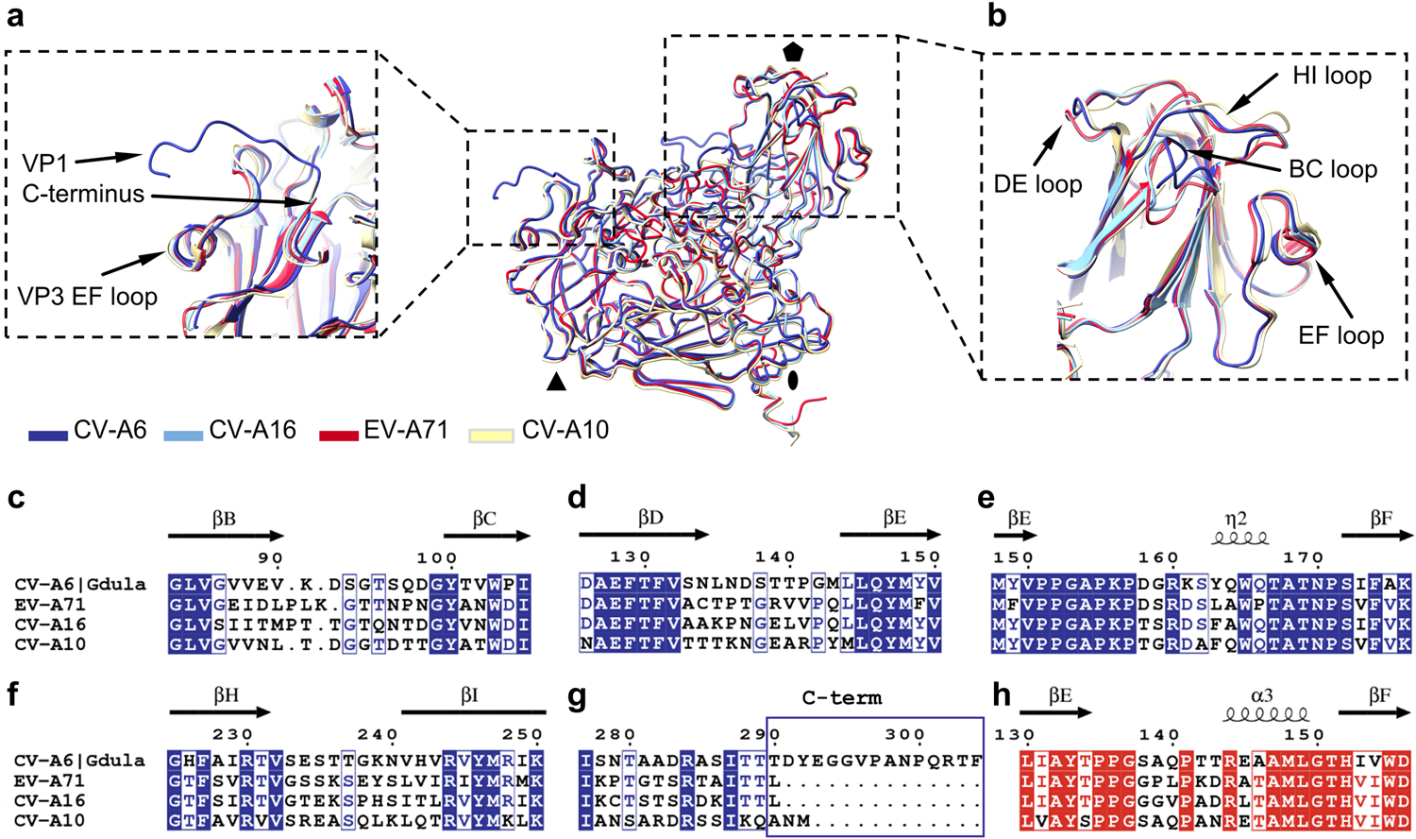
Comparison of structures of capsid protomers from virions of CV-A6, CV-A16, EV-A71, and CV-A10. The proteins are shown in cartoon representation. **a**, **b** Major differences between viruses were found in VP1 C-termini and VP1 surface loops positioned near the fivefold symmetry axis. **a** CV-A6 virion is unique in containing an exposed C-terminus of VP1, which covers the EF loop of VP3. **b** Detail of the differences in BC and HI surface loops of VP1. **c**–**h** Sequence alignment of VP1 (**c**–**g**, blue) and VP3 (**h**, red) from CV-A6, EV-A71, CV-A16, and CV-A10 showing the amino acid sequence variation of surface-exposed regions. The secondary structure elements of CV-A6 are indicated at the top.

The structure of the CV-A6 virion is most similar to those of CV-A16, EV-A71, and CV-A10. The viruses share more than 69% sequence identity, and the structures of their capsid protomers can be superimposed with an r.m.s.d. lower than 0.65 Å for at least 92% of the C^α^ atoms available for the comparisons (Supplementary Tables 4,  5). Because of their role in infection, virion structures enable the identification of antigenic epitopes for vaccine development. It has been shown that, among enteroviruses causing HFMD, the structural differences in the surface loops of VP1 give rise to the different antigenicity of these viruses^31,33,46,47^. Comparison of the structures of the VP1 loops of the CV-A6 virion with their equivalents in CV-A16, EV-A71, and CV-A10 shows variations in particular for the BC loop (r.m.s.d. of 0.52, 0.83, and 0.91 Å, respectively), and HI loop (r.m.s.d. of 0.88, 1.08, and 0.80 Å, respectively) (Fig. 4). In addition, the C-terminal arm of VP1 of the CV-A6 virion extends across the particle surface and covers the EF loop of VP3 (Fig. 4). The C-terminal segment of VP1 of CV-A6 has been shown to be antigenic, similar to that of CV-A10 ^33,46^. In contrast, the C-terminal arm is absent in the VP1 subunits of EV-A71 and CV-A16, explaining why in epitope studies their C-terminal peptides do not induce an immune response^31,33,48,49^. The differences in antigenicity-relevant determinants between the virion and altered particles suggest that the virion of CV-A6 confers a different immunogenicity. We anticipate that the availability of the virion structure in combination with the recent breakthrough in epitope prediction reliability, as shown for FMDV, EV-A71, CV-A16, and CV-A10 ^50,51^, will help to identify epitopes that CV-A6 shares with other enteroviruses, but also reveal differences that explain the low HFMD vaccine cross-protection.

### Capsid-genome contacts in the CV-A6 virion

**Fig. 5:**
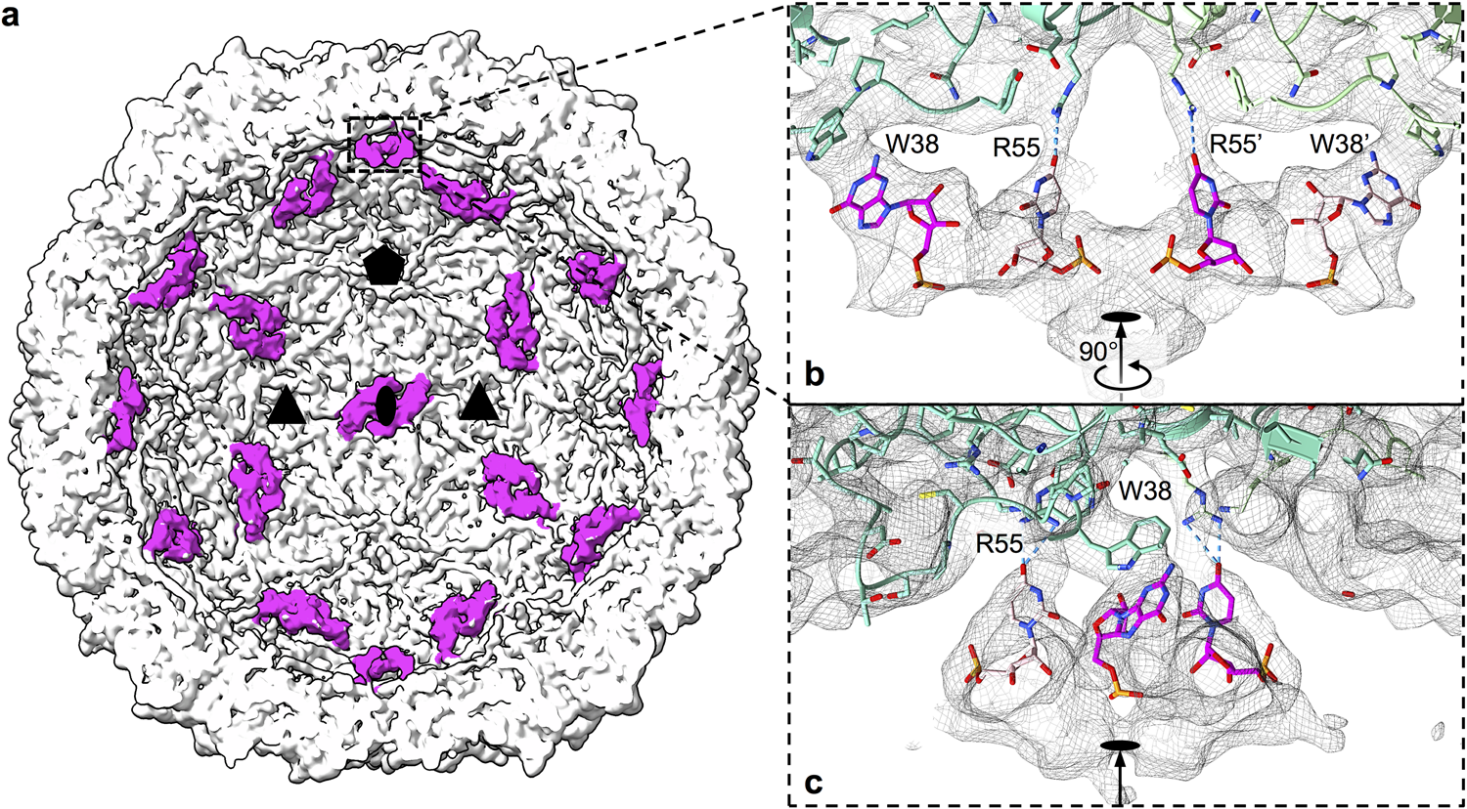
Genome-capsid contacts in CV-A6 virion. **a** Surface representation of cryo-EM reconstruction of CV-A6 virion with front half removed to show putative icosahedrally ordered RNA segments (magenta) located next to twofold symmetry axes. Oval, triangle, and pentamer icons indicate two-, three-, and fivefold icosahedral symmetry axes, respectively. **b**, **c** Mesh representation of density connecting putative RNA segments with side chains of Trp38 and Arg55 of VP2. The protein and RNA structures are shown in stick representation with carbon atoms of VP2 subunits distinguished by the hues of green. The carbon atoms of the two guanines and uracils modelled in the putative RNA density are shown in magenta and pink. Trp38 of VP2 is involved in a π–π stacking interaction with the guanine base. Arg55 of VP2 may form hydrogen bonds with uracil. The nucleotides are linked in a hexanucleotide and were refined against the shown density map using the software ChimeraX plugin ISOLDE^98^. **c** View perpendicular to **b**. For clarity, only the Trp38:guanine stacking interaction in front of the twofold axis is shown.

Cryo-EM reconstruction of the CV-A6 virion with imposed icosahedral symmetry reveals that parts of the virus RNA genome follow icosahedral symmetry and form contacts with the capsid (Fig. 5a). The reconstruction contains densities positioned next to the twofold axes of the capsid, which may correspond to RNA segments. The side chain of Trp38 of VP2 interacts at a distance of 4.6 Å with a density disc that is connected to the elongated curved putative RNA density positioned next to a twofold axis (Fig. 5b, c). In numerous other enterovirus structures, including poliovirus, Trp38 of VP2 or its homologues were shown to form putative π–π stacking interactions with bases from the RNA genome^52,53^. The base stacking against Trp38 in the CV-A6 virion was modelled as guanine for the best density match and the preference of guanine for tryptophan-mediated RNA π–π stacking interactions^54^. A second putative interaction between the RNA genome and the capsid is formed by Arg55 from VP2 (Fig. 5b). The positively charged side chain of arginine was shown to play a role in capsid-genome contacts, although the positions of arginines in the capsids of enteroviruses vary^52,53,55^. Arg55, which is coordinated by a cation–π stacking interaction with VP2 Tyr41, contacts the other end of the putative RNA density from Trp38. For example, Arg55 may form a hydrogen bond with a putative uracil base^56^ (Fig. 5b). No additional RNA interactions were observed in the CV-A6 virion. In contrast, Xu et al. (2017) suggested that the N-terminus of VP3 in the altered particle of CV-A6 interacts with the RNA^32^. Similar interactions can be observed in our structure of the altered particle of CV-A6 (Supplementary Fig. 8).

### Plaque-forming unit (PFU)-to-virion ratio of CV-A6

Here we show that the inoculum of CV-A6 strain Gdula contains virions, however, only 1.2% of the particles were virions, whereas the majority (91.4%) were altered particles, as identified by the classification procedures of the single-particle reconstruction pipeline (Supplementary Fig. 2).

To determine the PFU-to-virion ratio of CV-A6, we performed a fluorescence-based infection assay and quantified the total number of virus particles by measurements of UV absorbance and direct counting of the virus particles in cryo-electron tomograms. CV-A6 and other A strain enteroviruses are difficult to cultivate, and determining the infectious particle titre by plaque assay is challenging^57^. Therefore, we used an anti-VP2 antibody in a fluorescence-based infection assay to determine that the CV-A6 sample contained 9.0 × 10^10^ ± 4.1 × 10^10^ PFU mL^−1^ (Supplementary Fig. 9 and Table 2). The concentration of CV-A6 was 2.8 mg mL^−1^, using the A_260 nm_ absorption coefficient established for poliovirus^58^, which corresponds to 2.1 × 10^14^ genome-containing virus particles per millilitre. In contrast, the counting of particles in cryo-tomograms yielded a concentration of 3.6 × 10^15^ ± 1.3 × 10^15^ genome-containing particles per millilitre (Supplementary Fig. 9 and Table 2). The discrepancy in the particle concentrations determined using the two approaches may be caused by the use of the UV absorption coefficient established for poliovirus instead of CV-A6^58^, or by the enrichment or depletion of virus particles during the blotting step of cryo-ET sample preparation. The ratio of PFU to genome-containing particles, including both virions and altered particles, is 1:2300 based on the particle concentration determined by A_260 nm_ absorbance, and 1: 40,000 based on the counting of particles in cryo-tomograms. Xu et al. (2017) reported a 50% tissue culture infectious dose (TCID50/mg) of 3.6 × 10^7^, which corresponds to a PFU-to-particle ratio of 1: 2.1 × 10^6^. Our classification of images of genome-containing particles from electron micrographs used for the CV-A6 structure determination identified the ratio of virions to altered particles to be 1:75. Taken together, our results show that the PFU-to-virion ratio for CV-A6 strain Gdula is 1:30 when using UV absorbance to determine the number of particles, or 1:500 when counting the particles using cryo-ET. The PFU-to-virion ratios of enteroviruses are usually about 1:1000, indicating that the CV-A6 particle concentration determined by cryo-tomography may be a more reliable estimate^59^. The PFU-to-virion ratios of animal viruses range from 1: 10 for herpes simplex virus 1^60,61^, through 1:1000 for many picornaviruses, including poliovirus^59^, up to 1:40,000 for human alpha herpesvirus 3^62,63^.

**Table 2:** Ratio of PFU to virions and altered particles.

PFU	Absorbance A_260 nm_	Tomography	Ratio of genome-containing particles to virions	Ratio of PFU to genome-containing particles	Ratio of PFU to virions
mL^−1^	genome-containing particles mL^−1^	genome-containing particles mL^−1^	cryo-EM dataset	Abs. / tomogr.	Abs. / tomogr.
9.0 × 10^10^ ± 4.1 × 10^10^ range ^a^: 3.3 × 10^10^–2.6 × 10^11^	2.1 × 10^14^	3.6 × 10^15^ ± 1.3 × 10^15^ range ^b^: 2.0 × 10^15^ – 5.7 × 10^15^	75	1:2300 / 1:40,000	1:30 / 1:500

Mean ± SD, where available.

^a^*N* = 6, 10 replicates each.

^b^*N* = 8.

### Virions of CV-A6 are required for infection

It has been speculated by Xu et al. (2017) that CV-A6 strain TW-2007-00141 is exceptional among enteroviruses by utilising altered particles to infect cells. This hypothesis was based on the absence of virions and high abundance of the altered particles in purified infectious samples of CV-A6^32^. Our findings of the low abundance of CV-A6 virions among altered particles in the cryo-EM dataset suggest that the virions may have been missed in the previous study of CV-A6 strain TW-2007-00141^32^. Enteroviruses are in general highly robust and retain infectivity for prolonged periods of time at room temperature. The CV-A6 virion exhibits all the characteristic features that confer high stability to other enteroviruses. CV-A6 possesses similarly-sized protomer interfaces to related enteroviruses with an equivalent number of salt bridges and hydrogen bonds. CV-A6 furthermore contains a long pocket factor and an unrestricting small valine side chain of pocket residue 184 in VP1, both implicated in greater virus stability^64^. However, the above-discussed low occupancy of the pocket factor may be a cause of the observed instability of the CV-A6 virion, as it has been previously shown that the occupancy of the pocket plays an essential role in capsid stability^40,42^. The successful targeting of enteroviruses by capsid-binding inhibitors provides evidence that the binding and release of lipids to the hydrophobic pocket is a dynamic process^42,65^. Lipid factors and thermostability-conferring salt ions could have been lost during the virus purification.

It has been shown previously that the altered particles of poliovirus are four orders of magnitude less infectious than virions, indicating that the altered particles do not considerably contribute to the spread of the virus^66^. The altered particles of enteroviruses HRV2, CV-B1, and CV-A10 were shown to be unable to bind to the virus-specific receptors ICAM-1, CAR, and KREMEN1, respectively^67–69^. The PFU-to-virion ratio of CV-A6 is similar to that of other enteroviruses. Taken together, our and previous results suggest that the infection by CV-A6 is mediated by virions.

## Conclusions

Here we present high-resolution structures of the CV-A6 virion, altered particle and empty particle. We show that CV-A6 forms virions with characteristics similar to those of other enteroviruses. Our observations corroborate the presumption that enteroviruses possess two fundamental structural configurations: a compact virion with the RNA genome and a variety of altered (expanded or RNA-less) assembly and infection intermediates^30^. We provide evidence that the infection of CV-A6 is mediated by virions. The structure of the CV-A6 virion is crucial for future studies of the interactions with its host receptor and the mechanisms of cell entry and genome delivery. The virion structure enables the development of vaccines and capsid-binding inhibitors that will prevent CV-A6-mediated HFMD and its ensuing medical complications.

## Methods

### Organisms and strains

Human Coxsackievirus A6 (Gdula, GenBank accession number AY421764) (ATCC, VR-1801) was cultivated on human rhabdomyosarcoma (RD) cells (ATCC, CCL-136) at 37 ˚C in a humidified 5% CO_2_ atmosphere.

### Virus production and purification

CV-A6 infection in RD cells was allowed to progress for 6 days, and the tissue culture supernatant from 50 culture tissue plates with a diameter of 15 cm was precipitated by the addition of sodium chloride (final concentration 0.2 M) and polyethylene glycol 8000 (final concentration 10% w/v) and incubation at 10 ˚C overnight. The virus pellet was resuspended in 12 mL of buffer A (0.25 M sodium chloride, 0.25 M HEPES, pH 7.5). Magnesium chloride was added to the suspension (final concentration 5 mM) for nuclease digest at room temperature for 30 min (final concentration of DNase 10 μg mL^−1^ and RNase 15 μg mL^−1^). Trypsin was added, and the digest was incubated at 37 ˚C for 30 min and quenched by the addition of ethylene diamine tetra-acetic acid. Nonidet P40 was added to a final concentration of 1% v/v, and the virus particles were pelleted on a 2 mL 30% w/v sucrose cushion in buffer A (50.2 Ti rotor, Beckman Coulter). Pellets were resuspended in a total of 2 mL of cold buffer A and separated on a continuous tartrate-sucrose gradient (10-40% w/v) in the same buffer at 36,000×*g*, 10 ˚C for a minimum of 2 h (SW-41 rotor, Beckman Coulter). The virus-containing band was extracted using a syringe and needle. The solution was buffer-exchanged into phosphate-buffered saline (PBS) and concentrated to 2.5 mg mL^−1^ using ultrafiltration spin-columns (Amicon, Merck).

### Immunofluorescence assays to detect virus infection

Confluent RD cell monolayers in 24-well plates (Corning, #4441) were infected with serial dilutions of CV-A6 inoculum dissolved in 2 mL of medium per well. After 1 h, the inoculum was removed and replaced with fresh medium, and the infection was allowed to proceed for 6 h. Cells were fixed in 3% w/v formaldehyde for 15 min at room temperature, rinsed with 0.5 mL of PBS, and permeabilized using 0.5 mL of 0.5% v/v Triton X-100 in PBS for 5 min, then rinsed three times with PBS before adding 1% w/v BSA in PBS blocking solution. Primary rabbit anti-CV-A6-VP2 poly-clonal antibody (GeneTex, #GTX132347) was applied to the fixed cells, at a dilution of 1 µg mL^−1^ and incubated for 1 h at room temperature. Subsequently, the cells were rinsed three times with PBS before adding goat anti-rabbit IgG (H + L) cross-adsorbed secondary antibody, Alexa Fluor 488 (Thermo Fisher, #A-11008), at a dilution of 1 µg mL^−1^ at room temperature in the dark for 1 h. Cells were washed three times using PBS and kept at 4 ˚C overnight. Cells were further stained with DAPI hydrochloride (Thermo Fisher, #D1306) at a dilution of 2 µg mL^−1^ and imaged within 2 h using a confocal laser scanning microscope (Zeiss Axio Observer Z1 LSM800). Five nonoverlapping fields of view per well were acquired, and fluorescent cells were counted manually. Statistical and graphical analysis was performed using R version 3.5.1^70^. The 50% tissue culture infectious dose (TCID50/mg) was calculated according to the Behrens–Kärber method^71^.

### Cryo-EM sample preparation and data collection

Four microlitres of virus suspension with a virus concentration of 2.5 mg mL^−1^ (2.8 mg mL^−1^ for tomography) were applied onto a glow-discharged R2/1 Cu 300 mesh holey carbon-coated grid (Quantifoil, JenaBioSciences). Grids were plunge-frozen in liquid ethane after blotting using a Vitrobot Mark IV (Thermo Fisher Scientific) (chamber temperature: 20 °C; humidity: 100%; blotting paper: grade 595, 55/20 mm diameter, Ted Pella) and stored in liquid nitrogen. Cryo-electron micrographs were acquired using a Titan Krios (Thermo Fisher Scientific), equipped with a field emission gun operating at 300 kV. The microscope was fitted with a Falcon II direct electron detector (Thermo Fisher Scientific). Dose-fractionated data were collected in low-dose nanoprobe mode with parallel illumination. The total dose per acquisition was 44-48 electrons/Å^2^. A total of 9862 micrographs were collected as seven-frame movies at five acquisition areas per hole using the programme EPU (Thermo Fisher Scientific). The target defocus was set to range from −1 to −3 µm, and the nominal magnification was set to 75,000×, corresponding to a pixel size of 1.063 Å.

For tomography, tilt series from −45˚ to +45˚ were collected in a Tecnai F20 microscope equipped with an Eagle 4k (HS) CCD camera operating at 200 kV at a total dose of 120 electrons/Å^2^ at 50,000× magnification and a defocus of −5 µm.

### Image processing and three-dimensional reconstruction

For single-particle analysis, movie frames were aligned and averaged using MotionCorr^72^. Contrast transfer function (CTF) estimation was performed using the programme gCTF^73^. Micrographs with excessive drift or astigmatism were excluded from the dataset. An initial set of ~3000 particles were picked manually using e2boxer in EMAN2^74^ and used in model training for subsequent automatic picking using the programme crYOLO^75^. Particles were extracted using the programme RELION 3.1 at a box size of 512 × 512 pixels using the coordinates determined by crYOLO^75,76^. Multiple rounds of reference-free two-dimensional (2D) classification were performed in RELION 3.1 on binned particles (box size 128 px). Genome-containing particles were distinguished from empty particles by their high intensity (bright) particle interior, indicating the presence of the RNA genome. Initial 3D models for each subset of particles (genome-containing, empty) were generated using stochastic gradient descent with imposed icosahedral symmetry in RELION 3.1. After 3D auto-refinement and several rounds of 3D classification without alignment in RELION 3.1, final particle subsets were selected. The genome-containing virions differed from the altered particles by their smaller particle radius and VP4 density. Particles were re-extracted and recentred in a final box size of 512 px, and further subjected to iterative cycles of 3D auto-refinement, Ewald sphere correction, post-processing, and CTF refinement in RELION 3.1. For third and fourth order aberration estimation, the group of altered particles was used, because it contained the most particles of the highest resolution. Micrographs were divided into four optical groups, based on acquisition area, and third and fourth order aberrations were refined using RELION 3.1. The resulting Zernike polynomial coefficients (optical group parameters) were assigned to the corresponding micrographs of the smaller particle sets of the empty particles and virions. A detailed overview of the 3D-reconstruction process workflow for each particle conformation is given in Supplementary Fig. 2. For the sharpening of the final maps of the virion, altered particle, and empty particle, B-factors of −33, −75 and −70 Å^2^ were applied, respectively. Local resolution estimation was performed using the programme MonoRes^77^, and a local resolution-based sharpened map was generated using the programme LocalDeblur^78^ implemented in Xmipp/Scipion^79,80^. For electron tomography, image alignment, volume reconstruction and nonlinear anisotropic diffusion filtering were performed in IMOD^81^.

### Structure building and refinement

To minimise computational load during structure refinement, cryo-EM density maps of the virion, altered and empty CV-A6 were reoriented to standard orientation with the icosahedral twofold symmetry axes aligned to Cartesian coordinate axes, and cropped using EMAN2^74^. CCP4 maprot and mapmask^82^ were used to move the origin of the map to the particle centre and to normalise the maps. The maps were assigned crystallographic P23 symmetry (CCP4 ncsmask, mapmask), in which each of the 12 crystallographic asymmetric units comprises five icosahedral asymmetric units (capsid protomers).

Initial atomic structure models of all three particles were generated by homology modelling upon CV-A6 (PDB 5XS4^32^) and rigid-body fitting of the models into the density maps using the programme Chimera version 1.2.5^83^. VP4 of the CV-A6 virion was manually built in COOT version 0.9.5^84^. The structures were refined in iterative cycles of interactive building in COOT, real-space refinement with added hydrogens in PHENIX version 1.19.2-4148^85^, and reciprocal space structure regularisation without hydrogens in REFMAC5^86^. Per-residue real-space correlation coefficients were calculated using phenix.real_space_correlation. Overall model-to-map correlation coefficients were calculated using phenix.map_model_cc. Final models were validated in MolProbity^87^ and with the PDB validation service (https://validate-pdbe.wwpdb.org/).

### Structure interpretation and data visualisation

Pairwise structural alignments were prepared using TopMatch (beta) (https://topmatch.services.came.sbg.ac.at/topmatch-search.html) with visual assessment in TopMatch_web^88,89^. Per-residue r.m.s.d. of C^α^ atoms was calculated with LSQKAB^90^. Structure superpositions of loop regions were performed in ChimeraX. Protein interfaces were analysed using PDBePISA (https://www.ebi.ac.uk/pdbe/pisa/)^91^. Sequence alignments were calculated using the programme MAFFT and visualised in ESPript3^92,93^. We used the colour-deficiency-friendly palettes roma and RdYlBu^94,95^ for the surface rendering of structure models. Figures were prepared using the programmes ChimeraX^83,96^ and Fiji/ImageJ^97^.

### Reporting summary

Further information on research design is available in the Nature Research Reporting Summary linked to this article.

## Supplementary information

Supplementary Information

Reporting summary

## Data Availability

Cryo-EM maps and structure coordinates were deposited with the following accession numbers: virion of coxsackievirus A6: Electron Microscopy Data Bank (EMD) EMD-14186 and PDB 7QW9; coxsackievirus A6 altered particle: EMD-14183, PDB 7QVX; coxsackievirus A6 natural empty particle EMD-14184, PDB-7QVY.
