# Supplementary material for: Cryo-electron microscopy and image classification reveal the existence and structure of the coxsackievirus A6 virion
Source: Commun Biol. 2022 Sep 2;5:898. doi: 10.1038/s42003-022-03863-2 (PMC9438360; doi:10.1038/s42003-022-03863-2)
Supplement: Supplementary file 1 — Supplementary Information [file 42003_2022_3863_MOESM1_ESM.pdf]

Supplementary Information for:

Cryo-electron microscopy and image classification reveal the existence and structure of the coxsackievirus A6 virion

Carina R. Büttner [1], Radovan Spurný [1], Tibor Füzik [1] & Pavel Plevka[1*]

1 Central European Institute of Technology - Masaryk University, Structural Biology, Structural Virology, Kamenice 5, 62500 Brno, Czech Republic

Correspondence to: Pavel Plevka, pavel.plevka@ceitec.muni.cz, +420 549 49 7756

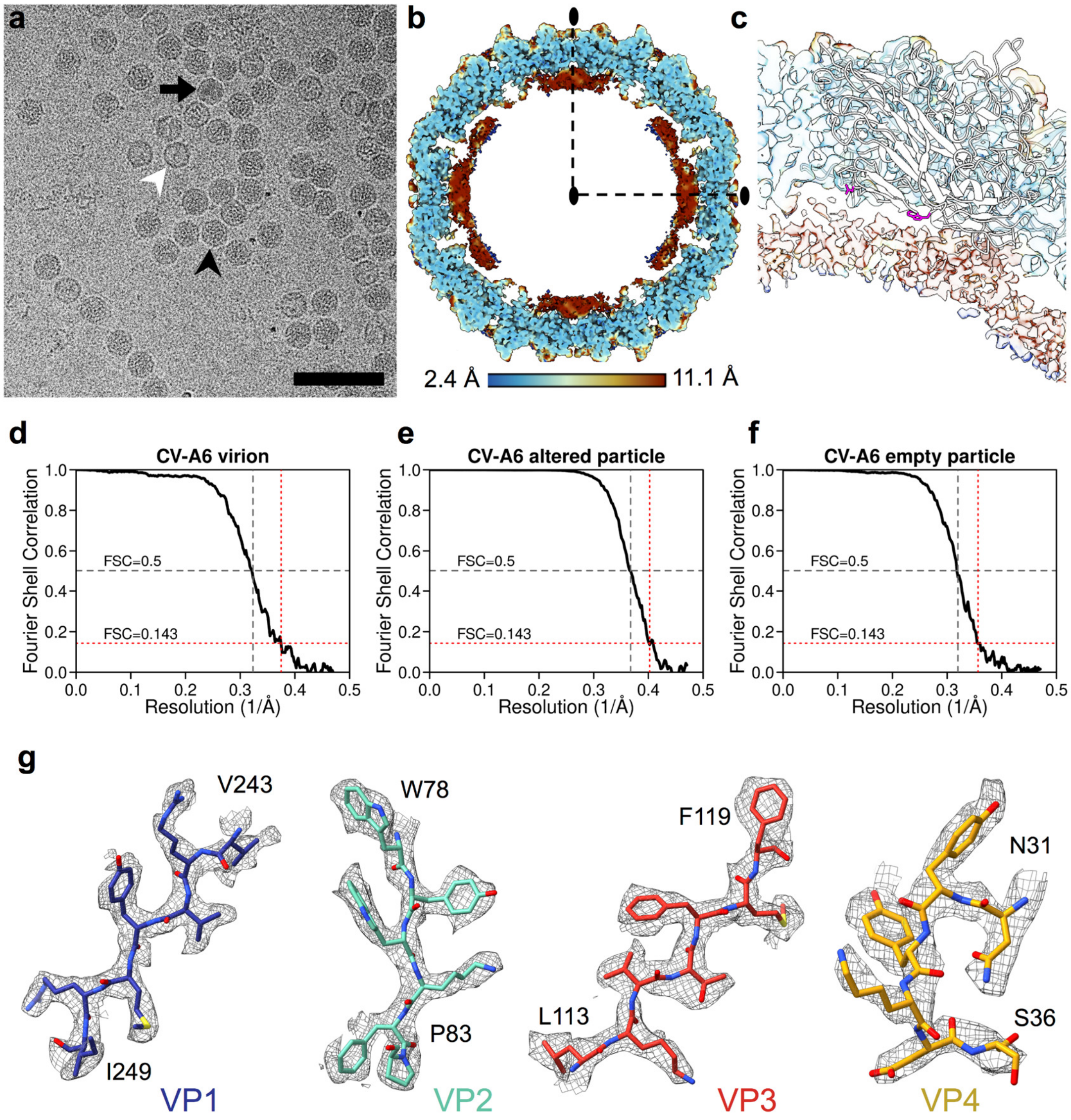

Supplementary Figure 1 Quality of cryo-EM data and reconstructions. **a** Representative cryo-electron micrograph of CV-A6 dataset containing altered (black arrowhead), empty particles (white arrowhead), and virions (black arrow). Scale bar 100 nm. **b** 20 Å-thick map slice showing local resolution variation (estimated by MonoRes) in reconstruction of CV-A6 virion. The map includes the capsid and outer layer of the RNA genome. **c** Detail of local resolution map around icosahedral asymmetric unit. The RNA-contacting residues Trp38 and Arg55 of VP2 are shown as magenta sticks. **d-f** Gold standard FSC curves of final density maps of CV-A6 virion (**d**), altered (**e**), and empty particle (**f**). **g** Fit of atomic structures of representative segments of VP1, VP2, VP3 and VP4 into corresponding densities. The proteins are shown as stick representation, and the map as grey mesh.

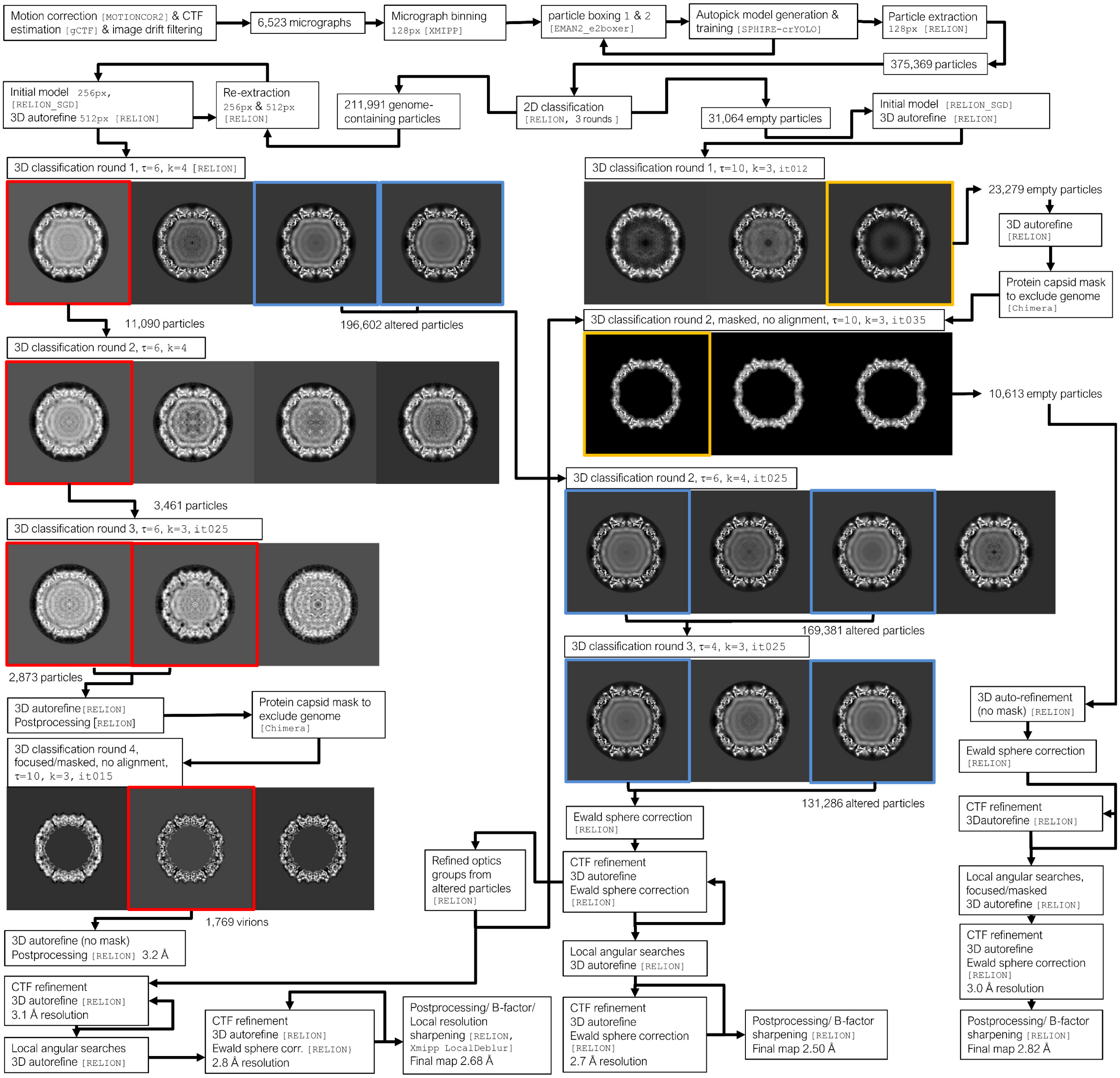

Supplementary Figure 2 Workflow of cryo-EM reconstruction of CV-A6 particles with details of the identification of the subset of CV-A6 virions. Genome-containing particles were distinguished from empty particles during 2D classification by their high intensity (bright) particle interior indicating the presence of the RNA genome. Multiple rounds of 3D classification of the genome-containing particles further distinguished virions from altered particles by their smaller radius and VP4 density located on the inside of the capsid. Each step lists the procedure and the number of used particles. Red, blue and yellow boxes indicate selected sets of virions, altered and empty particles, respectively, used in further analyses.

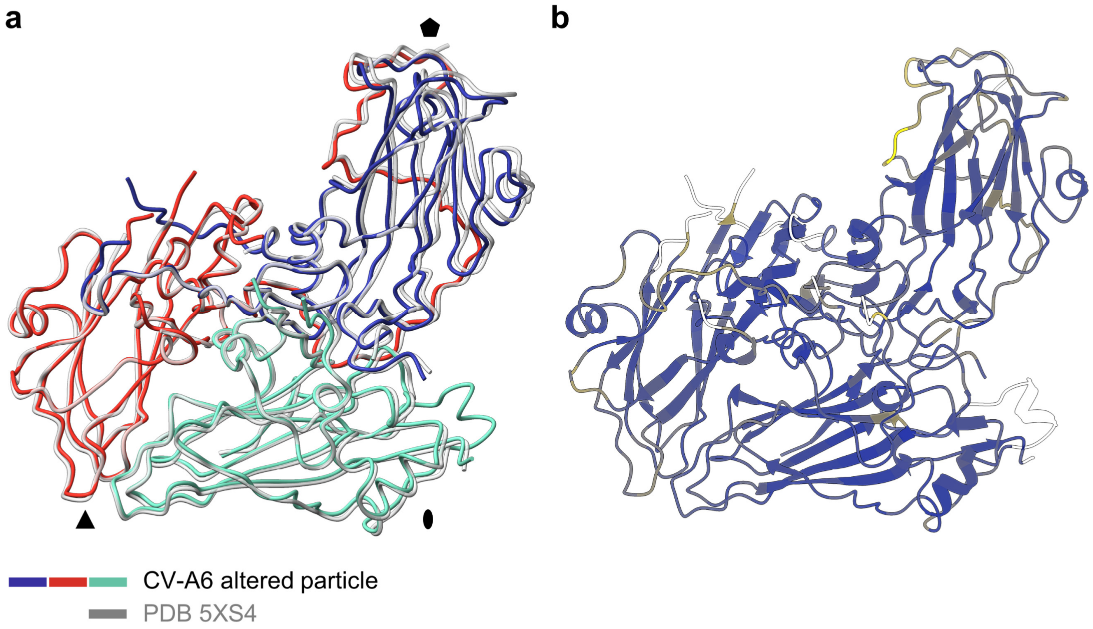

Supplementary Figure 3 Comparison of the icosahedral asymmetric units of altered particles of CV-A6 strains Gdula and TW-2007-00141. **a** Superposed protomers of the altered particle from this study (coloured) with the previously reported altered particle (grey, PDB 5XS4)[1]. Capsid protein chains are depicted as ribbon diagrams and coloured according to the convention: VP1 (blue), VP2 (green), VP3 (red). Positions of the twofold, threefold and fivefold icosahedral symmetry axes are indicated with an oval, triangle, and pentagon, respectively. **b** Structural deviations (r.m.s.d.) between altered particle determined in this study and PDB 5XS4 mapped onto the protomer from this study. Regions of low deviation (r.m.s.d. ≤ 3 Å) are in blue, to yellow (≤ 4 Å). Chain segments without matching residues are in white.

VP1

```
                α1                                  βA  η1      α2          α3
Gdula   .DPISNAIENAVSTLADTTISRVTAANTAASSHSLGTGRVPALQAAETGASSNASDENLIETRCVMNRNGVNEASVEHFY
TW2007  NDPIANAVESAVSALADTTISRVTAANTTASTHSLGTGRVPALQAAETGASSNASDENLVETRCVMNRNGVNEASVEHFY

          βB            βC          α4        βD              βE
Gdula   SRAGLVGVVEVKDSGTSQDGYTVWPIDVMGFVQQRRKLELSTYMRFDAEFTFVSNLNDSTTPGMLLQYMYVPPGAPKPDG
TW2007  SRAGLVGVVEVKDSGTSLDGYTVWPIDVMGFVQQRRKLELSTYMRFDAEFTFVSNLSNSTTPGMLLQYMYVPPGAPKPDG

          η2        βF      βG1       βG2                             βH
Gdula   RKSYQWQTATNPSIFAKLSDPPPQVSVPFMSPASAYQWFYDGYPTFGEHKQATNLQYGQCPNNMMGHFAIRTVSESTTGK
TW2007  RKSYQWQTATNPSVFAKLSDPPPQVSVPFMSPATAYQWFYDGYPTFGEHKQATNLQYGQCPNNMMGHFAIRTVSESTTGK

          βI1                    βI2
Gdula   NVHVRVYMRIKHVRAWVPRPFRSQAYMVKNYPTYSQTISNTAADRASITTTDYEGGVPANPQRTF
TW2007  NVHVRVYMRIKHVRAWVPRPLRSQAYMVKNYPTYSQTITNTATDRASITTTDYEGGVPASPQRTS
```

VP2

```
                 βA1    βA2        η1                     η2    βB          βC
Gdula   SPSVEACGYSDRVAQLTVGNSTITTQEAANIVLSYGEWPGYCPSTDATAVDKPTRPDVSVNRFYTLSTKSWKTESTGWYW
TW2007  SPSVEACGYSDRVAQLTVGNSTITTQEAANIVLSYGEWPEYCPSTDATAVDKPTRPDVSVNRFYTLSTKSWKTESTGWYW

          η3      α1         βD              βE1        βE2            α2       βF1
Gdula   KFPDVLNDTGVFGQNAQFHYLYRSGFCMHVQCNASKFHQGALLVVVIPEFVVAASSPATKPNGQGLYPDFAHTNPGKEGQ
TW2007  KFPDVLNDTGVFGQNAQFHYLYRSGFCMHVQCNASKFHQGALLVAAIPEFVIAASSPATKPNGRGLYPDFAHTNPGKDGQ

                     η4  βF2      βG1       βG2      βH
Gdula   VFRDPYVLDAGIPLSQALVFPHQWINLRTNNCATIIMPYVNALPFDSALNHSNFGLAVIPISPLKYCNGATTEVPITLTI
TW2007  EFRDPYVLDAGIPLSQALVFPHQWINLRTNNCATIIMPYVNALPFDSALNHSNFGLVVIPISPLKYCNGATTEVPVTLTI

        βI
Gdula   APLNSEFSGLRQAIKQ
TW2007  APLNSEFSGLRQAIKQ
```

VP3

```
                                                  βA1  α1   βA2         η1   βB
Gdula   GLPTELKPGTNQFLTTDDGTSPPILPGFEPTPLIHIPGEFTSLLDLCRIETILEVNNTTGTTGVNRLLIPVRAQNNVDQL
TW2007  GFPTELKPGTNQFLTTDDGTSPPILPGFEPTPLIHIPGEFTSLLDLCQIETILEVNNTTGTIGVSRLLIPVRAQNNVDQL

        βC            η2   α2   βD1   βD2         βE             α3   βF
Gdula   CASFQVDPGRNGPWQSTMVGQICRYYTQWSGSLKVTFMFTGSFMATGKMLIAYTPPGSAQPTTREAAMLGTHIVWDFGLQ
TW2007  CASFQVDPGRNGPWQSTMVGQICRYYTQWSGSLKVTFMFTGSFMATGKMLIAYTPPGSAQPATREAAMLGTHIVWDFGLQ

          βG1        βG2   η3      βH             βI1        βI2
Gdula   SSVTLVIPWISNTHFRAVKTGGVYDYYATGIVTIWYQTNFVVPPDTPSEANIIALGAAQENFTLKLCKDTDEIRQTAEYQ
TW2007  SSVTLVIPWISNTHFRAVKIGGVYDYYATGIVTIWYQTNFVVPPDTPTEANIIALGAAQKNFTLKLCKDTDEIQQTAAYQ

Gdula   N
TW2007  .
```

VP4

```
                                         η1          α1
Gdula   GAQVSAQKSGTHETGNIATEGSTINFTNINYYKDSYAASASRQDFTQDPTKFTSPVLDAIKEAAAPLQ
TW2007  GAQVSTQKSGSHETRNVATEGSTINFTNINYYKDSYAASASRQDFAQDPAKFTRPVLDTIREVAAPLQ
```

Supplementary Figure 4 Structure-based pairwise sequence alignment of capsid proteins VP1-4 from strain CV-A6 Gdula (GenBank accession number AY421764; used in this study) to TW-2007-00141 (KR706309) [1]. Secondary structure elements of the CV-A6 virion (this study) are depicted above the sequence. α-helices, 3_{10}-helices and β-strands are indicated with Greek letters α, η, and β, respectively. Beta-strands are named according to convention for the single jelly-roll fold proteins containing two antiparallel four-stranded β-sheets BIDG and CHEF.

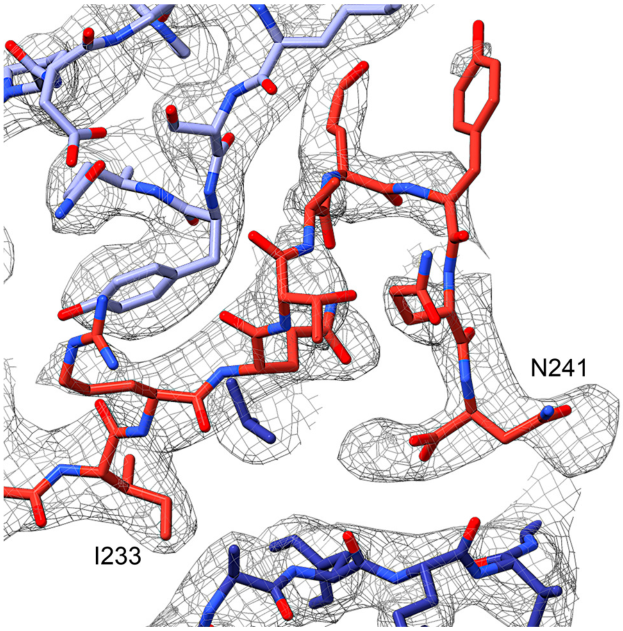

Supplementary Figure 5 Additional C-terminal residue N241 of VP3. Mesh representation of the locally sharpened density of the CV-A6 virion around the C-terminus of VP3 in red, neighbouring VP1 subunits in shades of blue. VP3 comprises an additional C-terminal amino acid residue due to an alternative (downstream) proteolytic cleavage site between VP3 and VP1 in the precursor polyprotein.

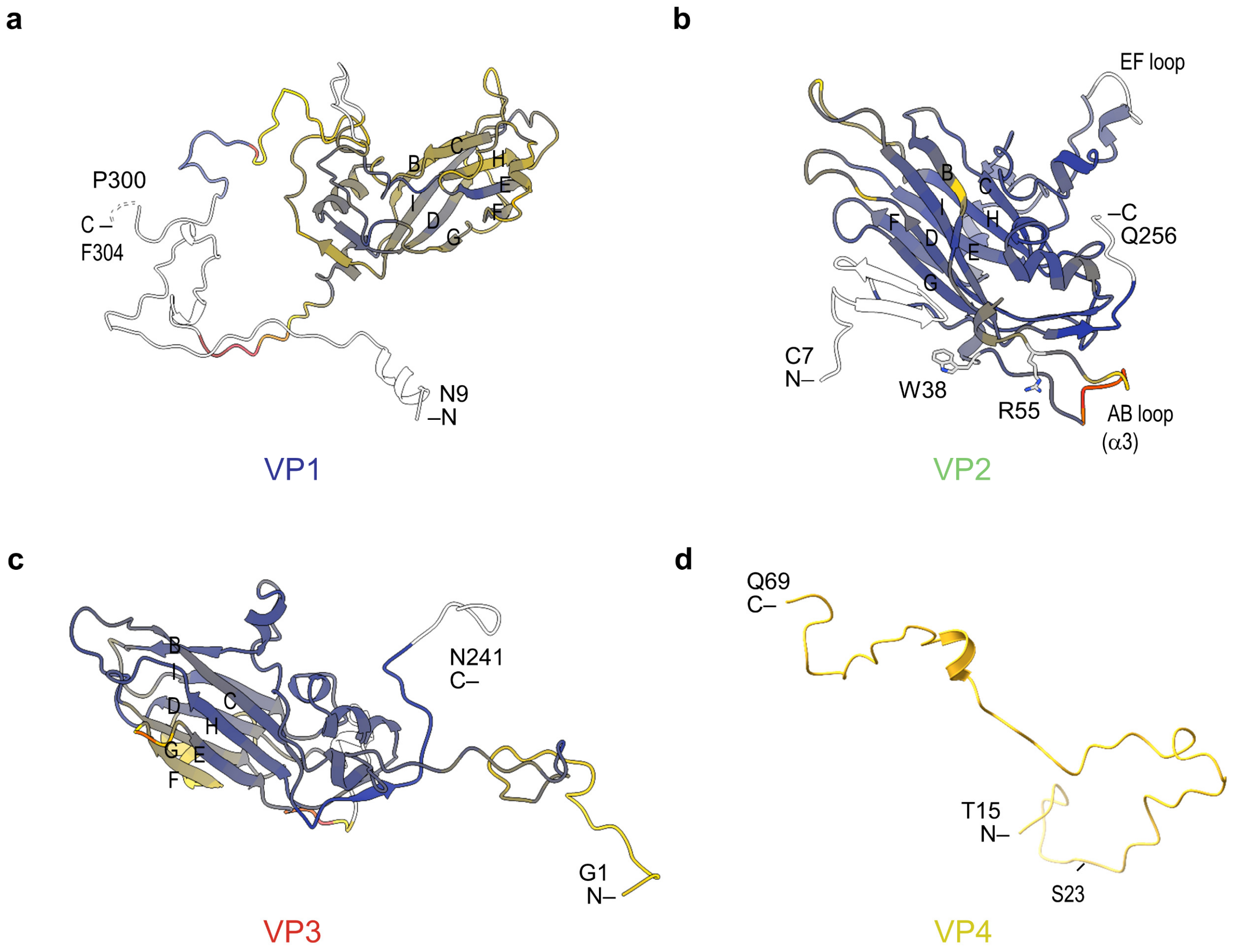

Supplementary Figure 6 Structural deviations between virion and altered particle of CV-A6. **a-c** R.m.s. deviations between the virion and altered particle are mapped onto the ribbon representations of individual coat proteins of the virion. Regions of low deviation (r.m.s.d. ≤ 3 Å) are in blue, through yellow (≤ 4 Å), to orange (≤ 6 Å) and dark red (> 6 Å). Missing chain segments are in white. β-strand identifiers of the jelly-roll CHEF and BIDG sheets are indicated. VP4 is omitted from the comparisons shown in panels **a-c**, because it is not structured and may be absent from the altered particle. **d** Structure of VP4 in the CV-A6 virion coloured according to convention in yellow.

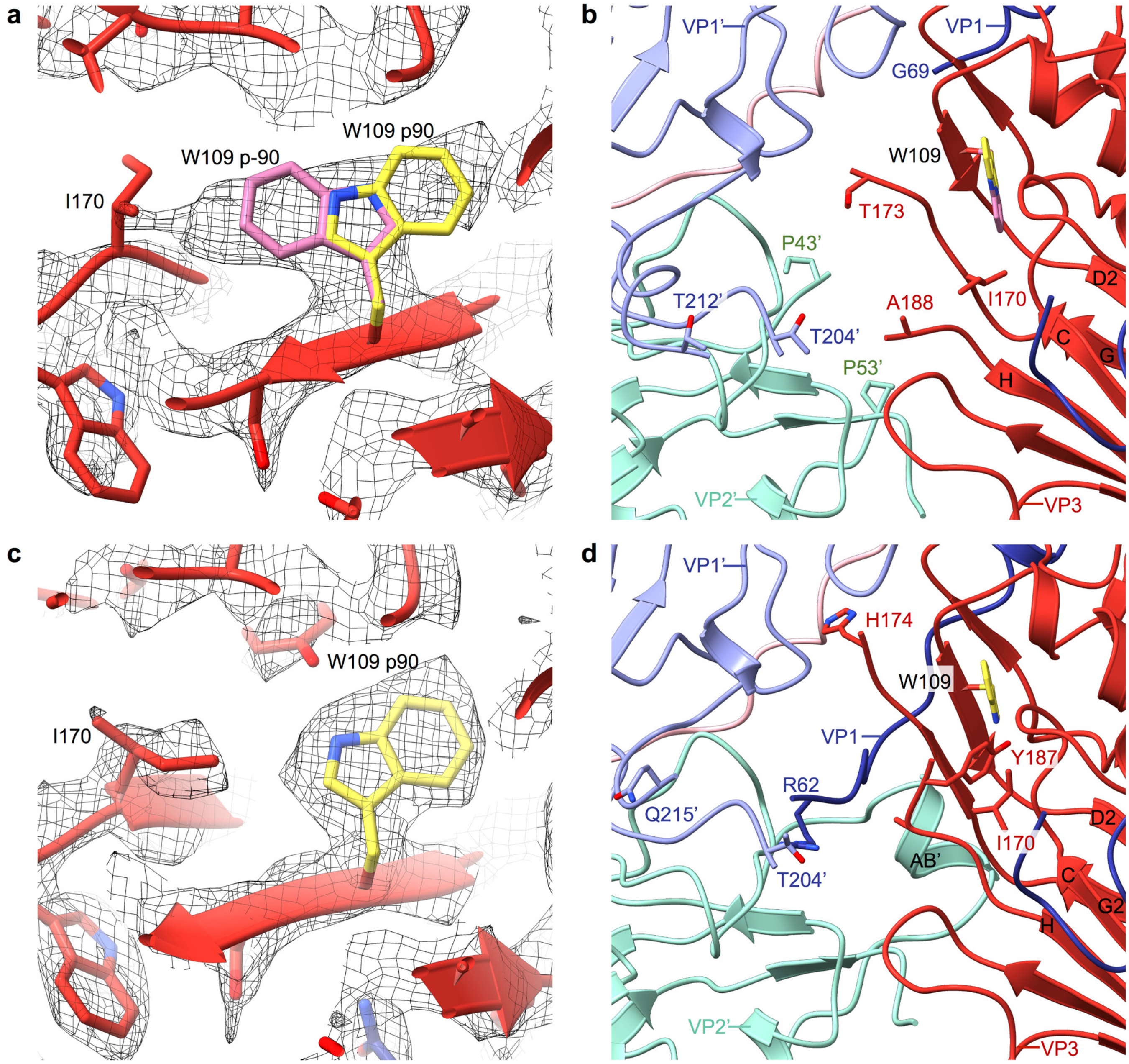

Supplementary Figure 7 Alternative conformations of Trp109 of VP3 in the empty particle of CV-A6. **a** The side chain of Trp109 of VP3 in the empty particle adopts two alternative rotamer conformations (p90, p-90), yellow and pink stick models, respectively. The structure of VP3 is shown in cartoon representation and residue side chains in stick representation, the normalised cryo-EM map is shown as a grey mesh at a contour level of 1.6 sigma (the same as in panel **c**). **b** Trp109 of VP3 is located in the CD loop, close to the GH loop of VP3, and at the interprotomer cleft. The interface is more flexible in the empty particle than in the altered particle of CV-A6, as indicated by the absence of resolved structures of the GH loop of VP3, and loops GH of VP1 and AB of VP2 from a neighbouring pentamer. The protein structures are shown in cartoon representation, the side chains

of the last resolved residues from loops of the capsid proteins are shown as stick models. VP1 is coloured in blue, VP2 in green, and VP3 in red. **c-d** Altered particle structure, views as in **a** and **b**. **c** Trp109 of VP3 adopts only one conformation in the altered particle of CV-A6. The side chain of Ile170 occupies the volume taken up by one of the alternative Trp109 conformers present in the empty particle. **d** The inter-protomer interface of the altered particle contains more resolved residues of the major capsid proteins than that of the empty particle.

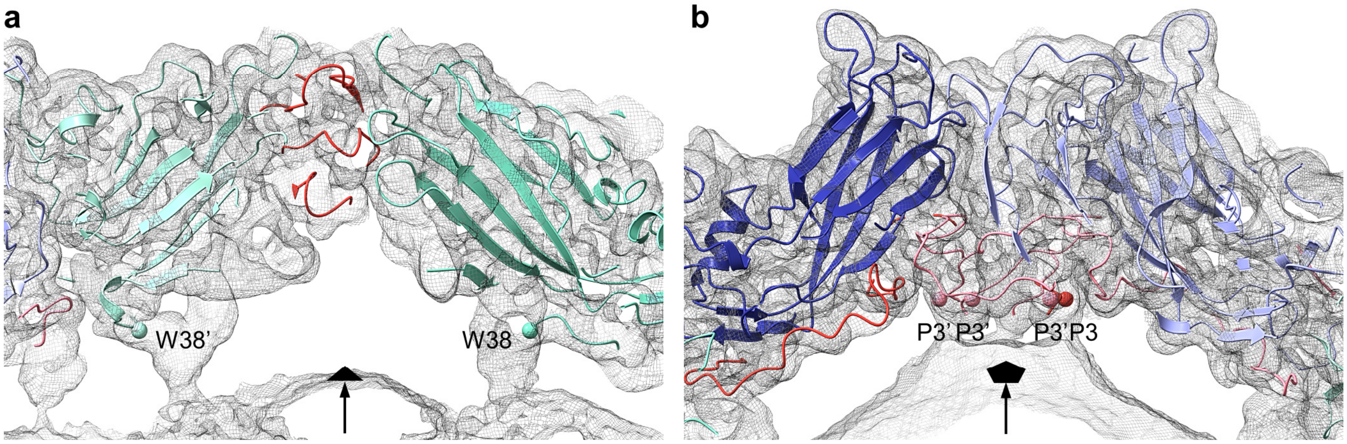

Supplementary Figure 8 Genome-capsid contacts in the CV-A6 altered particle. Mesh representation of the density of the CV-A6 altered particle connecting the putative RNA segments with the interacting residues (spheres). **a** Capsid-RNA interactions mediated by Trp38 of VP2. The green balls indicate the position of Trp38. **b** Capsid-RNA interactions mediated by the N-termini of VP3. The red balls indicate the position of Pro3. Triangle and pentamer icons and arrows indicate the directions of the three- and fivefold icosahedral symmetry axes, respectively. View perpendicular to the indicated symmetry axes. The protein structures are shown in cartoon representation with capsid protein subunits distinguished by shades of green and blue.

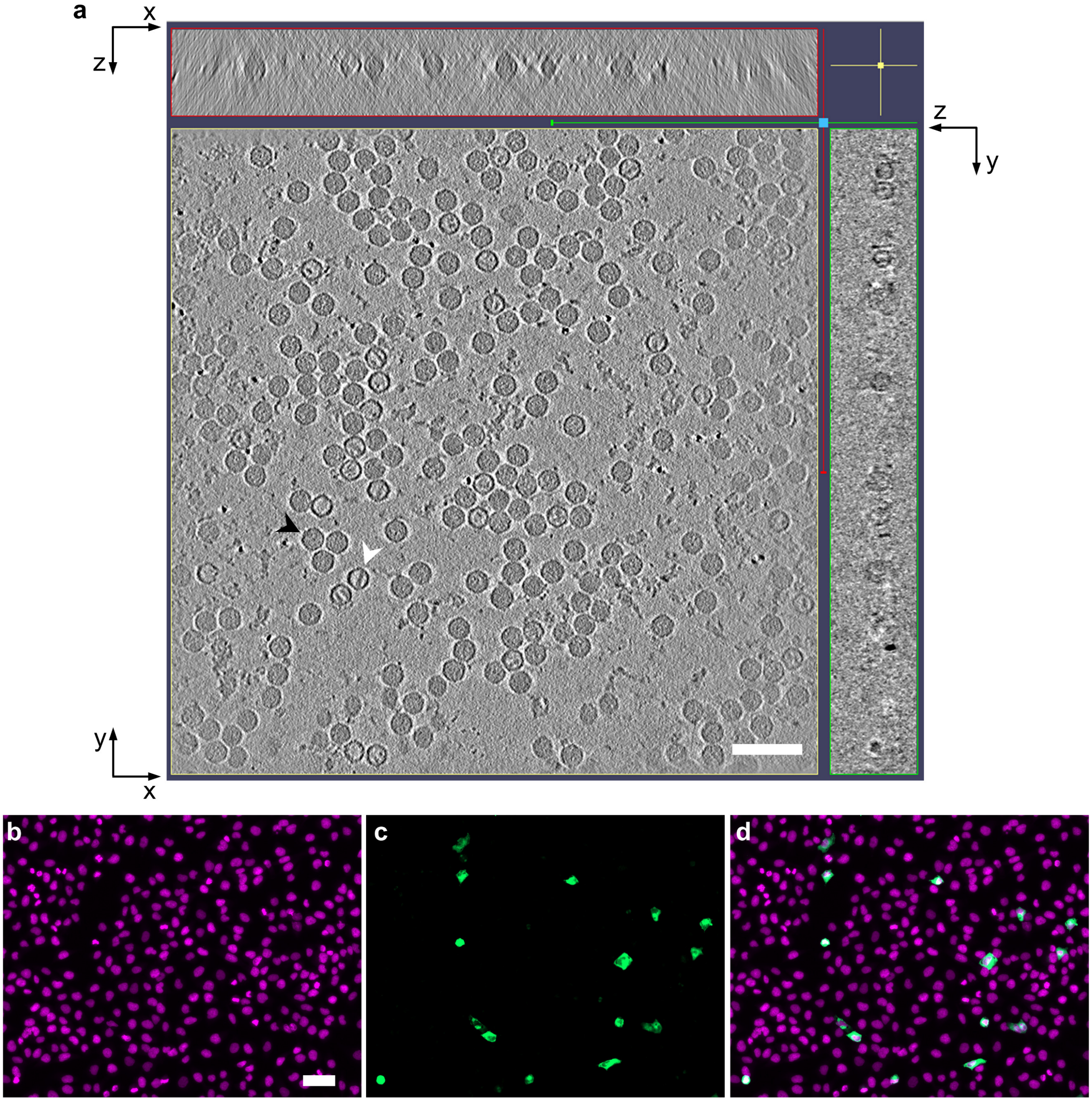

Supplementary Figure 9 Determination of CV-A6 PFU-to-particle ratio. **a** Cryo-electron tomogram (displayed using the XYZ window in IMOD/etomo) for counting genome-containing (black arrowhead) and empty particles (white arrowhead). Scale bar 100 nm. **b-d** Representative immunofluorescence microscopy image of CV-A6-infected RD cells. Single cells can be distinguished by nucleic acids labelling using DAPI (magenta, **b**), infected cells were labelled with CV-A6 anti-VP2 antibody and secondary DyLight 488 conjugate antibody (green, **c**), merge (**d**). Each infected cell counted towards one infectious virion. Scale bar 100 µm.

Supplementary Table 1 Structure comparison (r.m.s.d., pairwise alignment) of CV-A6 particles						
		VP1	VP2	VP3	VP4	Protomer / Total
CV-A6 virion : CV-A6 altered particle	Aligned Cα	193 (63)	204 (80)	191 (79)	-	548 (83) / 659
	r.m.s.d. (Å)	1.14	0.60	0.82	-	0.82
CV-A6 virion : CV-A6 empty particle	Aligned Cα	202 (66)	201 (79)	189 (78)	-	540 (84) / 641
	r.m.s.d. (Å)	1.22	0.61	0.82	-	0.82
CV-A6 altered : CV-A6 empty particle	Aligned Cα	214 (70)	206 (80)	214 (89)	-	633 (99) / 638
	r.m.s.d. (Å)	0.24	0.36	0.29	-	0.31
CV-A6 altered : CV-A6 expanded (PDB 5XS4)	Aligned Cα	210 (69)	203 (79)	208 (86)	-	621 (100) / 623
	r.m.s.d. (Å)	0.61	0.48	0.67	-	0.73
CV-A6 empty : CV-A6 procapsid (PDB 5XS5)	Aligned Cα	211 (69)	192 (75)	192 (80)	-	594 (97) / 610
	r.m.s.d. (Å)	0.59	0.52	0.65	-	0.75

The number in parentheses indicates the respective percentage of available amino acid residues used for the r.m.s. deviation calculations. Superpositions were performed using the USCF ChimeraX [2] tool matchmaker with the default cutoff distance of 2.0 Å for the inclusion of residues.

Supplementary Table 2 Resolved residues of CV-A6 virion, altered and empty particles				
Coat protein residues		Virion	Altered particle	Empty particle
VP1 1-304 [a]		9-300, 304 [c]	62-204, 215-292	69-204, 212-252
	missing	1-8, 301-303	1-61, 205-214, 293-304	1-68, 205-211, 253-304
VP2 1-256		7-256	30-138, 144-251	30-43, 53-137, 145-251
	missing	1-6	1-29, 139-143, 252-256	1-29, 44-52, 138-144, 252-256
VP3 1-241 [b]		1-241	1-174, 187-233	1-173, 188-232
	missing	-	175-186, 234-241	174-187, 233-241
VP4 1-69		15-69	-	-
	missing	1-14	-	-

[a,b] VP1 has been renumbered Asp-1 to Phe-304, as VP3 contains an additional C-terminal residue due to a downstream cleavage site between VP3 and VP1.
[c] Density visible for aromatic side chain of Phe-304.

Supplementary Table 3 Pocket factor binding site residues in CV-A6 virion	
Capsid subunit	Amino-acid residues
VP1	Ile-105, Asp-106, Val-107, Met-108, Phe-129, Phe-131, Tyr-147, Tyr-149, Ile-173, Val-184, Val-186, Met-189, Tyr-195, Trp-197, Asn-222, Met-224, Phe-227, Asn-269
VP3	Ile-24

**Supplementary Table 4 Protein structure similarity for CV-A6 virion by chain-based one-against-all searches[a] in TopMatch**				
**Capsid protein**		**1st-ranked species**	**2nd-ranked species**	**3rd-ranked species**
**VP1**		Coxsackievirus A10	Coxsackievirus A16	Enterovirus A71
	PDB	6SMG	5C4W	3ZFE
	Z score	29.46	29.14	28.86
	Aligned Cα	270	269	264
	r.m.s.d. (Å)	0.88	0.95	0.88
**VP2**		Enterovirus A71	Coxsackievirus A16	Coxsackievirus A10
	PDB	4C0U	5ABJ	6SMG
	Z score	25.49	25.46	25.09
	Aligned Cα	244	244	237
	r.m.s.d. (Å)	0.79	0.79	0.48
**VP3**		Coxsackievirus A10	Coxsackievirus A16	Enterovirus A71
	PDB	6SMG	5ABJ	6Z3Q
	Z score	2.94	22.84	22.31
	Aligned Cα	236	236	235
	r.m.s.d. (Å)	0.56	0.70	0.96
**VP4**		Coxsackievirus A9	Echovirus 7	Poliovirus 1
	PDB	1D4M	3IYP	3J8F
	Z score	19.31	18.92	18.53
	Aligned Cα	52	51	53
	r.m.s.d. (Å)	1.70	1.67	1.92

[a] PDB version as of 2021-09-08

**Supplementary Table 5 Structure comparison (r.m.s.d., pairwise alignment) of CV-A6 virion with related enteroviruses**						
		**VP1**	**VP2**	**VP3**	**VP4**	**Protomer / Total**
CV-A6 virion : EV-A71 virion (PDB 3VBS)	Aligned Cα	242 (81)	238 (94)	222 (92)	36 (52)	751 (87) / 813
	r.m.s.d. (Å)	0.61	0.46	0.54	0.66	0.58
CV-A6 virion : CV-A16 virion (PDB 5C4W)	Aligned Cα	255 (86)	237 (93)	228 (95)	35 (51)	754 (87) / 814
	r.m.s.d. (Å)	0.66	0.45	0.46	0.66	0.56
CV-A6 virion : CV-A10 virion (PDB 6AKS)	Aligned Cα	256 (86)	236 (93)	232 (97)	42 (61)	764 (89) / 811
	r.m.s.d. (Å)	0.67	0.47	0.55	0.77	0.65
CV-A6 virion : Poliovirus 1 virion (PDB 1ASJ)	Aligned Cα	212 (70)	207 (80)	213 (89)	35 (51)	667 (77) / 799
	r.m.s.d. (Å)	0.81	0.57	0.71	0.90	0.74

The number in parentheses indicates the respective percentage of available amino acid residues used for the r.m.s. deviation calculations. Superpositions were performed using the USCF ChimeraX [2] tool matchmaker with the default cutoff distance of 2.0 Å for the inclusion of residues.

Supplementary References

1. Xu, L., *et al.* Atomic structures of Coxsackievirus A6 and its complex with a neutralizing antibody. *Nature Communications* **8**, 505 (2017).

2. Pettersen, E. F., *et al.* UCSF ChimeraX: Structure visualization for researchers, educators, and developers. *Protein Science* **30**, 70-82 (2021).
