# Supplementary material for: Cryo-electron microscopy and image classification reveal the existence and structure of the coxsackievirus A6 virion
Source: Commun Biol. 2022 Sep 2;5:898. doi: 10.1038/s42003-022-03863-2 (PMC9438360; doi:10.1038/s42003-022-03863-2)
Supplement: Supplementary file 2 — Reporting summary [file 42003_2022_3863_MOESM2_ESM.pdf]

nature portfolio

Corresponding author(s): Pavel Plevka

Last updated by author(s): Jul 29, 2022

Reporting Summary

Nature Portfolio wishes to improve the reproducibility of the work that we publish. This form provides structure for consistency and transparency in reporting. For further information on Nature Portfolio policies, see our Editorial Policies and the Editorial Policy Checklist.

Statistics

For all statistical analyses, confirm that the following items are present in the figure legend, table legend, main text, or Methods section.

n/a	Confirmed	
☐	☒	The exact sample size (*n*) for each experimental group/condition, given as a discrete number and unit of measurement
☐	☒	A statement on whether measurements were taken from distinct samples or whether the same sample was measured repeatedly
☒	☐	The statistical test(s) used AND whether they are one- or two-sided<br>*Only common tests should be described solely by name; describe more complex techniques in the Methods section.*
☒	☐	A description of all covariates tested
☐	☒	A description of any assumptions or corrections, such as tests of normality and adjustment for multiple comparisons
☐	☒	A full description of the statistical parameters including central tendency (e.g. means) or other basic estimates (e.g. regression coefficient) AND variation (e.g. standard deviation) or associated estimates of uncertainty (e.g. confidence intervals)
☒	☐	For null hypothesis testing, the test statistic (e.g. *F*, *t*, *r*) with confidence intervals, effect sizes, degrees of freedom and *P* value noted<br>*Give P values as exact values whenever suitable.*
☒	☐	For Bayesian analysis, information on the choice of priors and Markov chain Monte Carlo settings
☒	☐	For hierarchical and complex designs, identification of the appropriate level for tests and full reporting of outcomes
☐	☒	Estimates of effect sizes (e.g. Cohen's *d*, Pearson's *r*), indicating how they were calculated

Our web collection on statistics for biologists contains articles on many of the points above.

Software and code

Policy information about availability of computer code

Data collection	Cryo-electron micrographs were recorded in EPU v1.12.<br>Tecnai TEM Imaging & Analysis embedded software Tomography v4 were used for acquisition of cryo-electron tomographic tilt series.<br>Fluorescence microscopy images were recorded in ZEN 2.3 (blue edition) software.
Data analysis	Single particle reconstructions and analysis were performed using gCTF v1.6, MotionCor2, RELION v3.1., and MonoRes and LocalDeblur implemented in Xmipp/Scipion v2.0. Reconstruction and non-linear anisotropic diffusion filtering of tomograms were performed in IMOD v. 4.9.12, and 4.10.45. R project v3.5.1 was used for descriptive statistics calculations. Fluorescence images were analyzed in ImageJ v1.53f51.

For manuscripts utilizing custom algorithms or software that are central to the research but not yet described in published literature, software must be made available to editors and reviewers. We strongly encourage code deposition in a community repository (e.g. GitHub). See the Nature Portfolio guidelines for submitting code & software for further information.

Data

Policy information about availability of data

All manuscripts must include a data availability statement. This statement should provide the following information, where applicable:

- Accession codes, unique identifiers, or web links for publicly available datasets
- A description of any restrictions on data availability
- For clinical datasets or third party data, please ensure that the statement adheres to our policy

Cryo-EM maps and structure coordinates were deposited with the following accession numbers: virion of coxsackievirus A6: Electron Microscopy Data Bank (EMD) EMD-14186 and PDB 7QW9; coxsackievirus A6 altered particle: EMD-14183, PDB 7QVX; coxsackievirus A6 natural empty particle EMD-14184, PDB-7QVY.

Field-specific reporting

Please select the one below that is the best fit for your research. If you are not sure, read the appropriate sections before making your selection.

☒ Life sciences ☐ Behavioural & social sciences ☐ Ecological, evolutionary & environmental sciences

For a reference copy of the document with all sections, see nature.com/documents/nr-reporting-summary-flat.pdf

Life sciences study design

All studies must disclose on these points even when the disclosure is negative.

Sample size	Sample sizes were not predetermined based on statistical methods, but were chosen according to the standards of the field (at least three independent biological replicates for each condition in the infection assay). The specific staining of individual virus-infected cells using known markers and the low observed variability between stained samples gave sufficient statistics of the effect size of interest.<br>The sample size of eight tomograms used to determine the virus particle concentrations was based on the number of available reconstructed high-quality tomograms.
Data exclusions	We did not exclude any data from consideration.<br>For cryo-EM single particle analysis, all picked virus particles were used in the initial classification analyses, and particle selection during the reconstruction workflow, as detailed in Methods, cryo-EM section, and the accompanying Supplementary Information figure, was performed according to standards in the field.
Replication	Reported infection assay results were consistently reproduced across multiple experiments with replicates giving similar results.<br>Denovo recalculation of all cryo-EM reconstructions using then newer software version RELION 3.1 gave similar results.
Randomization	Randomization was not necessary as investigators studied macromolecular complexes under well controlled conditions. Bias during particle reconstructions is further minimized (applying the gold-standard approach in the field) by comparing two randomly split, independent halves of the datasets.
Blinding	n/a

Reporting for specific materials, systems and methods

We require information from authors about some types of materials, experimental systems and methods used in many studies. Here, indicate whether each material, system or method listed is relevant to your study. If you are not sure if a list item applies to your research, read the appropriate section before selecting a response.

Materials & experimental systems

n/a	Involved in the study
☐	☒ Antibodies
☐	☒ Eukaryotic cell lines
☒	☐ Palaeontology and archaeology
☒	☐ Animals and other organisms
☒	☐ Human research participants
☒	☐ Clinical data
☒	☐ Dual use research of concern

Methods

n/a	Involved in the study
☒	☐ ChIP-seq
☒	☐ Flow cytometry
☒	☐ MRI-based neuroimaging

Antibodies

Antibodies used	rabbit anti-CV-A6-VP2 poly-clonal antibody (GeneTex, #GTX132347, Lot No. 42335, RRID AB_2886624);<br>goat anti-rabbit IgG (H+L) cross-adsorbed secondary antibody, Alexa Fluor 488 (ThermoFisher, #A-11008, RRID AB_143165)
Validation	Western Blot; Based on [Manufacturer's] internal testing result, the anti-CV-A6-VP2 antibody may cross react with Coxsackievirus A16.

Eukaryotic cell lines

Policy information about cell lines

Cell line source(s)	RD (ECACC 85111502, ATCC CCL 136)
Authentication	*Describe the authentication procedures for each cell line used OR declare that none of the cell lines used were authenticated.*

Mycoplasma contamination

Confirm that all cell lines tested negative for mycoplasma contamination OR describe the results of the testing for mycoplasma contamination OR declare that the cell lines were not tested for mycoplasma contamination.

Commonly misidentified lines
(See ICLAC register)

Name any commonly misidentified cell lines used in the study and provide a rationale for their use.
